# Mitochondrial BK Channel Openers CGS7181 and CGS7184 Exhibit Cytotoxic Properties

**DOI:** 10.3390/ijms19020353

**Published:** 2018-01-25

**Authors:** Bartłomiej Augustynek, Piotr Koprowski, Daria Rotko, Wolfram S. Kunz, Adam Szewczyk, Bogusz Kulawiak

**Affiliations:** 1 Laboratory of Intracellular Ion Channels, Nencki Institute of Experimental Biology, 3 Pasteur St., 02-093 Warsaw, Poland; bartlomiej.augustynek@ibmm.unibe.ch (B.A.); p.koprowski@nencki.gov.pl (P.K.); d.rotko@nencki.gov.pl (D.R.); a.szewczyk@nencki.gov.pl (A.S.); 2 Institute of Biochemistry and Molecular Medicine, University of Bern, Bühlstrasse 28, CH-3012 Bern, Switzerland; 3 Department of Experimental Epileptology and Cognition Research and Department of Epileptology, University of Bonn, 25 Sigmund-Freud Strasse, D-53105 Bonn, Germany; wolfram.kunz@ukb.uni-bonn.de

**Keywords:** potassium channel openers, mitoBK_Ca_ channel, neuronal cells, cytotoxicity, mitochondria

## Abstract

Potassium channel openers (KCOs) have been shown to play a role in cytoprotection through the activation of mitochondrial potassium channels. Recently, in several reports, a number of data has been described as off-target actions for KCOs. In the present study, we investigated the effects of BK_Ca_ channel openers CGS7181, CGS7184, NS1619, and NS004 in neuronal cells. For the purpose of this research, we used a rat brain, the mouse hippocampal HT22 cells, and the human astrocytoma U-87 MG cell line. We showed that CGS7184 activated the mitochondrial BK_Ca_ (mitoBK_Ca_) channel in single-channel recordings performed on astrocytoma mitoplasts. Moreover, when applied to the rat brain homogenate or isolated rat brain mitochondria, CGS7184 increased the oxygen consumption rate, and can thus be considered a potentially cytoprotective agent. However, experiments on intact neuronal HT22 cells revealed that both CGS7181 and CGS7184 induced HT22 cell death in a concentration- and time-dependent manner. By contrast, we did not observe cell death when NS1619 or NS004 was applied. CGS7184 toxicity was not abolished by BK_Ca_ channel inhibitors, suggesting that the observed effects were independent of a BK_Ca_-type channel activity. CGS7184 treatment resulted in an increase of cytoplasmic Ca^2+^ concentration that likely involved efflux from internal calcium stores and the activation of calpains (calcium-dependent proteases). The cytotoxic effect of the channel opener was partially reversed by a calpain inhibitor. Our data show that KCOs under study not only activate mitoBK_Ca_ channels from brain tissue, but also induce cell death when used in cellular models.

## 1. Introduction

Potassium channel openers (KCOs) have been described as a group of synthetic chemicals activating different classes of potassium channels. Originally, KCOs were identified as activators of two types of plasma membrane K^+^ channels: ATP-regulated potassium (K_ATP_) channels and large-conductance calcium-activated potassium (BK_Ca_) channels [1].

A variety of different small synthetic molecules have been described as BK_Ca_ channel openers. Many of them are benzoimidazolone derivatives (e.g., NS1619 and NS004). Electrophysiological studies revealed that NS1619 activates plasma membrane BK_Ca_ channels and that this effect is suppressed by BK_Ca_ channel inhibitors such as iberiotoxin (IbTx) and paxilline (Pax) [2]. Another group of BK_Ca_ channel openers comprises the indole carboxylate compound CGS7181 and its analogues, such as CGS7184 [3]. Studies using the patch-clamp technique revealed that these chemicals induce a concentration-dependent stimulation of whole-cell BK_Ca_ currents in smooth muscle cells from several animal species [3]. Recently, other molecules such as 1,4-benzothiazine derivatives and NS11021 have been described as potent and specific activators of this channel [4,5]. Reports have also described a group of natural BK_Ca_ channel openers, such as terpene derivatives (e.g., pimaric acid and maxikdiol), flavonoids (e.g., apigenine, naringenin), and phenolic derivatives (e.g., magnolol) [6].

KCOs have recently attracted attention as potent therapeutic agents. It was found that pretreatment with certain potassium channel activators has cytoprotective effects against ischemia/reperfusion (IR) injury of cardiac and neuronal cells [1,7]. The detailed mechanism underlying these effects remains unknown. It was suggested that the cytoprotection phenomenon induced by KCOs might be mediated by potassium channels located in the inner mitochondrial membrane [8,9]. The mitoBK_Ca_ channel was first described in glioma cells using patch-clamp recordings [10]. Later on, the presence of the channel was confirmed in other tissues such as cardiac muscle [8,9], skeletal muscle [11], and the brain [12,13,14]. The basic pharmacological properties of mitoBK_Ca_ channels have been found to be similar to those of BK_Ca_ channels present in the plasma membrane [1]. It has been suggested that activation of the mitoBK_Ca_ by NS1619 leads to the cytoprotection of cardiomyocytes insulted during ischemia/reperfusion [8] or treated with ouabain [9]. Additionally, it has been reported that the activation of mitoBK_Ca_ channels with KCOs influences mitochondrial reactive oxygen species (ROS) production and this mechanism was postulated to be important for cytoprotection [15,16].

Apart from studies showing the direct activation of mitochondrial BK_Ca_ channels by KCOs, a number of reports described alternative sites of action for these compounds [1,17]. It has even been suggested that the observed neuroprotective effect of NS1619 may be due to off-target interactions rather than the modulation of mitochondrial potassium channels [18,19]. Alternative targets for NS1619 have also been previously reported in glioma cell mitochondria [20]. Additionally, it has been shown that CGS7184 induces calcium release from intracellular stores in endothelial cells [21,22] and initiates glial cell death [23]. These observations clearly show that KCOs have multiple targets within the cell.

Here, we report that CGS7181 and CGS7184 activate the mitoBK_Ca_ channel. Activation of the channel was observed directly on the level of single channels and indirectly by measuring the respiration rate in isolated mitochondria and brain homogenates. However, when used in neuronal cell cultures, these compounds show a strong cytotoxic effect, most likely due to the deregulation of calcium homeostasis and calpain activation.

## 2. Results

### 2.1. CGS7184 Activates mitoBK_Ca_

**Figure 1:**
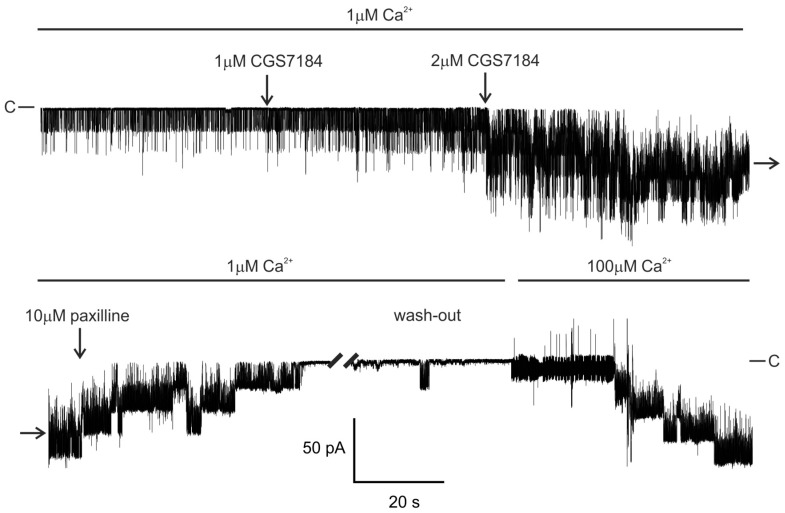
The mitochondrial BK_Ca_ channel is activated by CGS7184. A representative patch-clamp recording of the activity of BK_Ca_ channels detected in mitochondria of the U-87 MG cell line. Continuous recording from one patch at −60 mV pipette voltage is shown. C—indicates closed channel level. Opening of channels is visible as downward deflections from the closed channel level.

The presence of BK_Ca_ in mitochondria of the U-87 MG cell line is a well-established observation [24,25]. Here, we tested the impact of CGS7184 on this channel. We observed an increase of the mitoBK_Ca_ activity after the addition of 0.3–2 µM CGS7184 in the presence of 1 µM Ca^2+^ ions at negative pipette voltages, to a various degree in different mitochondrial membrane patches, possibly reflecting the intrinsic heterogeneity of the channel. An example of this activatory action of CGS7184 is shown in Figure 1.

In this case, channel open probability (NPo) increased from 0.09 in the control to 0.55 in the presence of 1 µM CGS7184, and to 3.07 in the presence of 2 µM CGS7184. This activity was subsequently blocked by 10 µM paxilline, a well-established alkaloid inhibitor of BK_Ca_ channels [2]. Paxilline block could be relieved by a wash-out with 100 µM but not 1 µM Ca^2+^, what is consistent with the mechanism of paxilline inhibition of BK_Ca_ channels, further supporting the notion that mitoBK_Ca_ was the target of these drugs [26]. On average, the open probability increased from 1.5 ± 3.5 in the control to 29.3 ± 27.3 (range 9.0 to 77.0) in the presence of 1 µM CGS7184 (*n* = 7).

**Figure 2:**
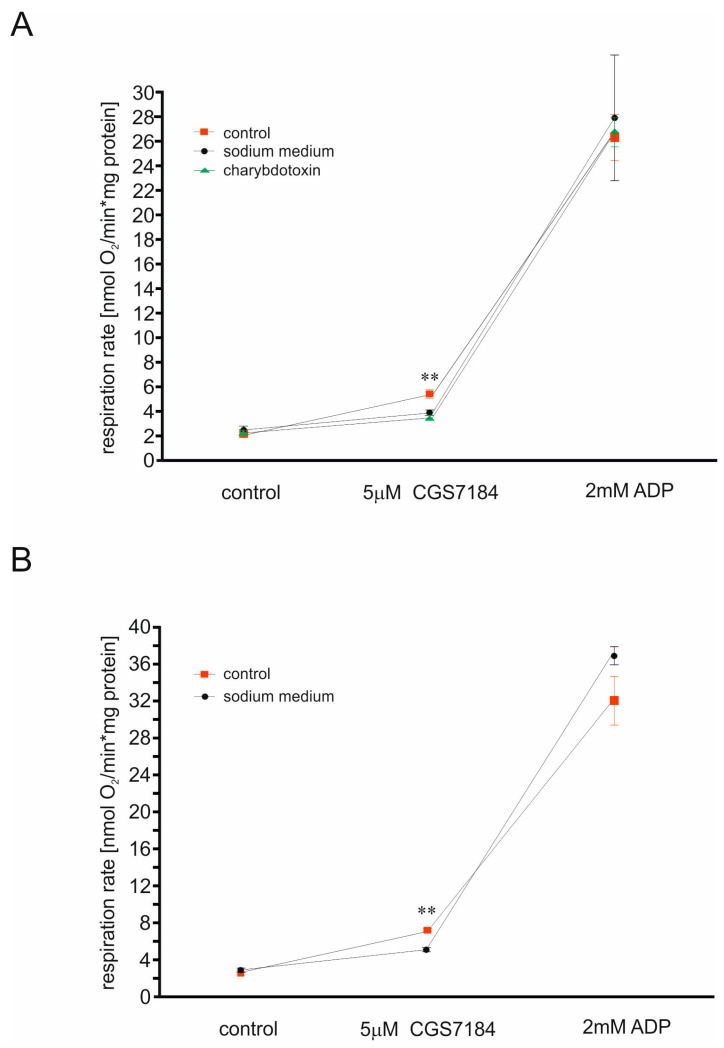
Potassium channel opener CGS7184 stimulates mitochondrial respiration via mitoBK_Ca_ opening. (**A**) Respiration rate measurements of isolated rat brain mitochondria upon the addition of 5 µM CGS7184 followed by the addition of 2 mM ADP. (**B**) Respiration rate measurement of isolated rat brain homogenate upon the addition of 5 µM CGS7184 followed by the addition of 2 mM ADP. ** *p* < 0.01 by one-way ANOVA followed by Tukey’s test.

Our previous data showed that CGS7184 reduced reactive oxygen species synthesis by isolated brain mitochondria via activation of the mitoBK_Ca_ channel [16]. Therefore, we used isolated rat brain mitochondria and rat brain homogenate to monitor the influence of CGS7184 on mitochondrial respiration. Application of 5 µM CGS7184 increased the respiration rate of both rat brain homogenate and isolated mitochondria (Figure 2). These effects were significantly reduced when potassium ions were replaced with sodium ions, or when BK_Ca_-type channel inhibitor was applied. However, in this experimental system, we used charybdotoxin instead of paxilline as the channel inhibitor, since paxilline has been shown to influence the function of heart and liver mitochondria independently of potassium ion fluxes [27].

### 2.2. CGS7181 and CGS7184 Induce HT22 Cell Death in a Dose- and Time-Dependent Manner

**Figure 3:**
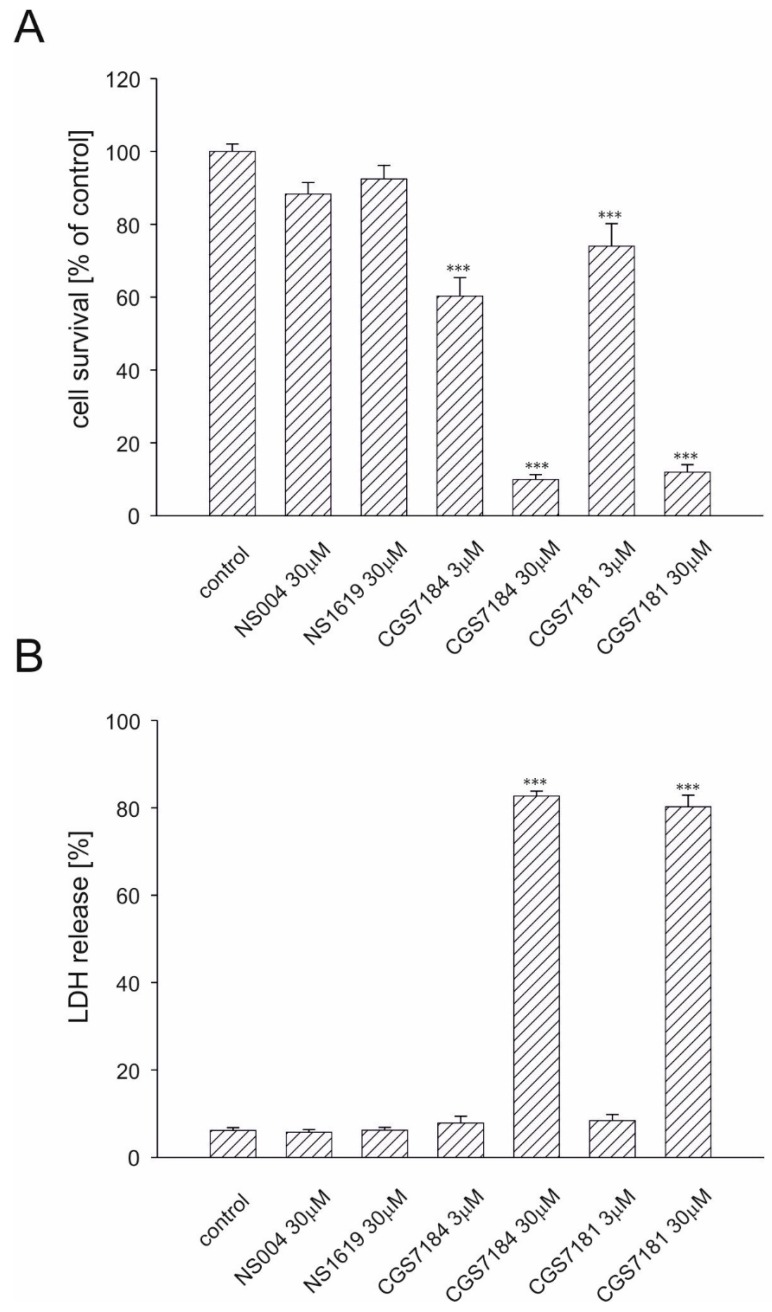
Potassium channel openers CGS7181 and CGS7184 induce HT22 cell death. HT22 cells were treated with potassium channel openers NS1619, NS004, CGS7181, and CGS7184. Cell survival was estimated with the MTT assay (**A**) or by LDH release (**B**) 18 h after insult. All data are expressed as means ± SEM from at least five independent experiments with at least three replicates per data point. *** *p* < 0.001 by one-way ANOVA followed by Tukey’s test.

Previous data proved that CGS7184 activates mitoBK_Ca_. However, treatment of HT22 cells, commonly used as a model for neuronal cell death, with this compound showed strong cytotoxic effects. Using the MTT (3-(4,5-dimethylthiazol-2-yl)-2,5-diphenyltetrazolium bromide) tetrazolium reduction assay, we found that after 18 h incubation, 3 µM CGS7181 induced toxicity in 26% of cells, whereas 3 µM CGS7184 induced toxicity in approximately 40% of cells. Incubation of HT22 cells with 30 µM CGS7181 or CGS7184 induced cell death in approximately 90% of the cells (Figure 3A). By contrast, neither NS1619 nor NS004 affected cell survival.

To further evaluate the toxic effect of tested compounds, we measured the amount of lactate dehydrogenase (LDH) released after treatment with KCOs (Figure 3B). Release of LDH into the incubation medium occurs as a result of cell membrane disruption during necrotic cell death. After 18 h of incubation of cells with NS1619 or NS004 (both at 30 µM), the level of LDH released was the same as in the control. Conversely, the incubation of HT22 cells with 30 µM CGS7181 or CGS7184 resulted in ca. 80% LDH release, which was in line with the results obtained by the MTT assay. On the other hand, release of LDH following treatment with 3 µM CGS7181 or CGS7184 was similar to that of the control cells. This could indicate that at low concentrations CGS7181 and CGS7184 may induce programmed cell death, whereas at higher concentrations, they lead to necrotic cell death and extensive cell membrane disruption.

**Figure 4:**
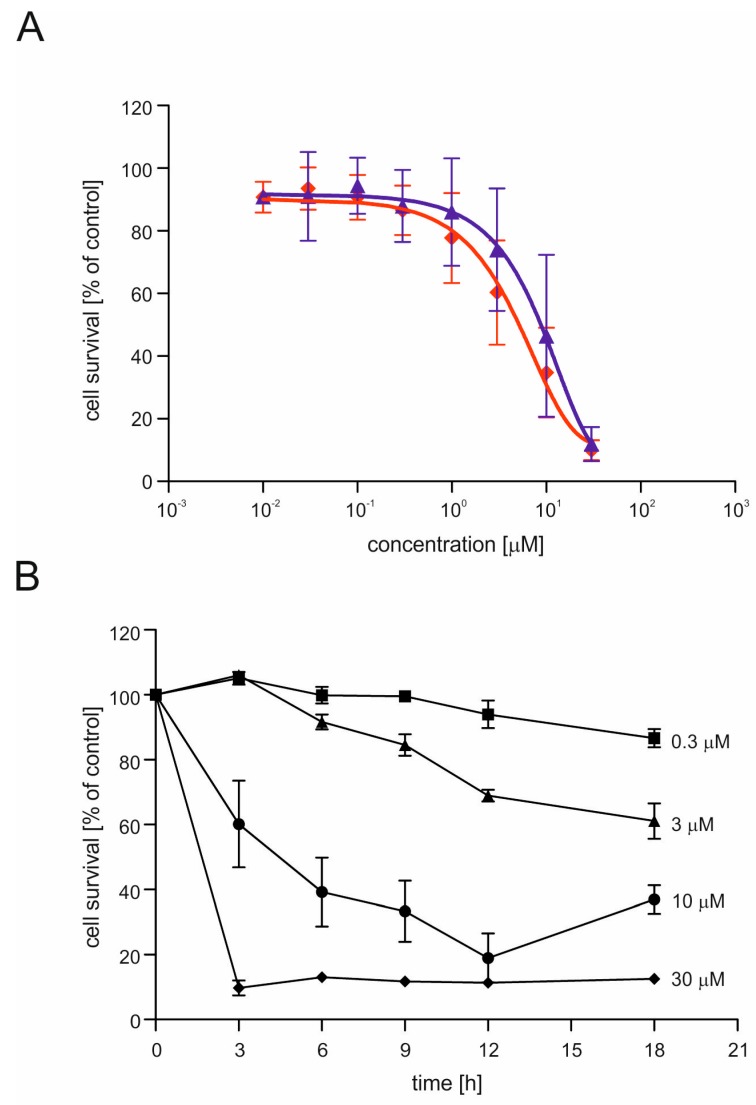

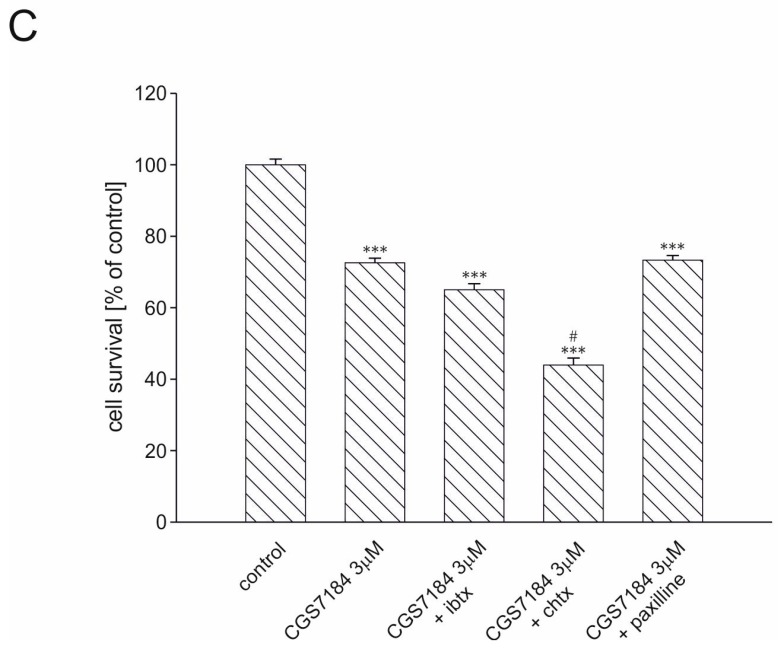
Dose and time dependence of toxic effects induced by CGS7181 and CGS7184. (**A**) HT22 cells were treated with potassium channel openers CGS7181 (diamonds) or CGS7184 (triangles). Cell survival was measured using the MTT assay 18 h after opener application. (**B**) HT22 cells were treated with the potassium channel opener CGS7184 at different concentrations: 30 µM (diamonds), 10 µM (circles), 3 µM (triangles), and 0.3 µM (squares). Cell survival was measured using the MTT assay 3, 6, 9, 12, and 18 h after opener application. (**C**) HT22 cells were treated with 3 µM CGS7184 in the presence of inhibitors of the BK_Ca_ channel. Cell survival was measured using the MTT assay 18 h after opener application. chtx-charybdotoxin (500 nM), ibtx-iberiotoxin (100 nM), pax-paxilline (2 µM). All data are expressed as means ± SEM from three independent experiments with three replicates per data point. # *p* < 0.05 vs. 3 µM CGS7184 and *** *p* < 0.001 vs. control by one-way ANOVA followed by Tukey’s test.

The toxic effects of CGS7181 and CGS7184 were dose dependent (Figure 4A). Next, we verified the time dependence of CGS7184 toxicity using the MTT assay (Figure 4B). High doses of CGS7184 rapidly induced cell death. For example, after 12 h, treatment with 10 µM of CGS7184 resulted in the death of almost 80% of the cells, while 3 h of incubation with 30 µM CGS7184 resulted in the death of over 90% of the cells.

### 2.3. BK_Ca_ Channels Are Not Involved in CGS7184-Induced HT22 Cell Death

As a next step, we wanted to investigate whether the observed toxic effects were due to the activity of BK_Ca_ channels. To this end, we preincubated HT22 cells for 30 min with BK_Ca_ channel inhibitors: 500 nM charybdotoxin, 100 nM iberiotoxin, or 4 µM paxilline. After this preincubation, 3 µM CGS7184 was added. The BK_Ca_ channel inhibitors were present throughout the entire period of incubation with KCOs. Eighteen hours after the addition of CGS7184, cell survival was measured by the MTT assay. We found that BK_Ca_ channel inhibitors did not block the CGS7184-induced toxicity (Figure 4C). Furthermore, in the presence of 500 nM charybdotoxin, we observed a stronger toxic effect of 3 µM CGS7184. These results suggest that the observed toxicity was related to non-specific activity of the potassium channel openers.

### 2.4. CGS7184 Elevates Cytoplasmic Calcium Concentration

**Figure 5:**
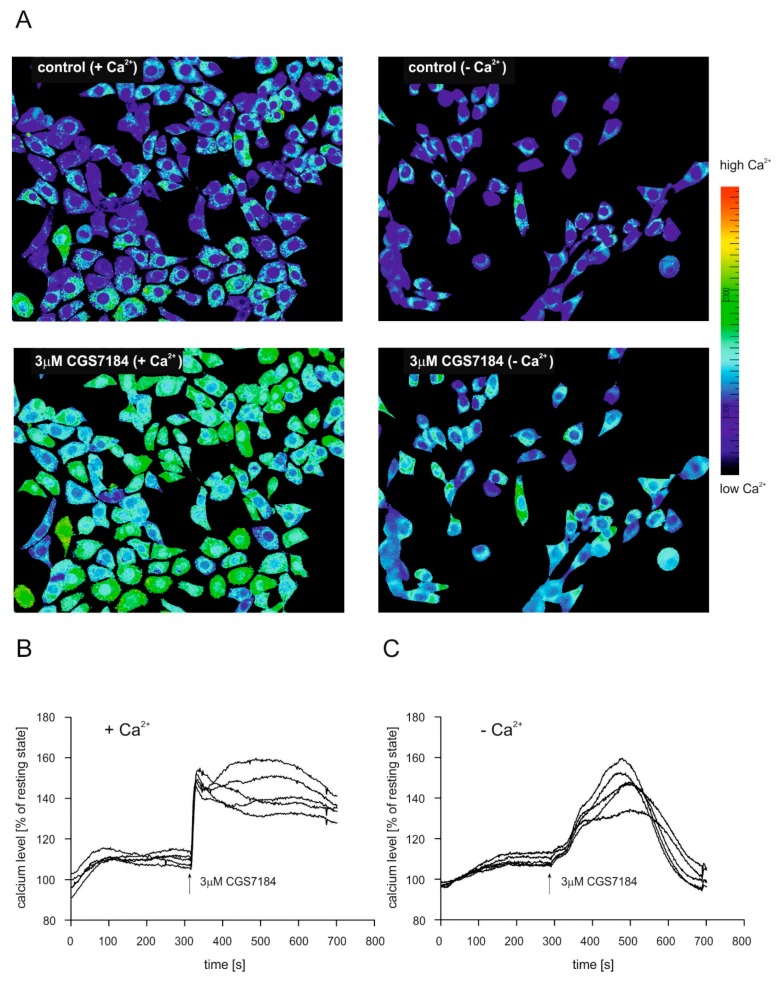
CGS7184 induces rapid increase in the cytoplasmic Ca^2+^ concentration. Calcium transients in single cells induced by 3 µM CGS7184 were recorded with fluorescent microscopy. HT22 cells were loaded with calcium sensitive dye FURA-2 AM prior to the experiment. (**A**) Representative images of calcium concentration levels are shown in before (control) and 3 min after application of the potassium channel opener (3 µM CGS7184), 20× magnification. (**B**) Quantification of cytosolic of calcium level changes measured in single cells in the presence of calcium ions in the external medium. (**C**) Quantification of cytosolic calcium level changes measured in single cells in the absence of calcium ions in the external medium.

Because deregulation of calcium homeostasis plays an important role in different cell death pathways, we decided to explore whether CGS7184 could alter calcium concentration levels in HT22 cells. Figure 5 presents time-lapse measurements of calcium concentration changes in single cells using fluorescence microscopy and calcium sensitive dye FURA-2. Figure 5A presents typical images of the FURA-2 signal ratio before (control) and after the addition of the channel opener (3 µM CGS7184). Initial experiments were performed in the medium containing 1.2 mM calcium ions (panel A “+Ca^2+^” and panel B). On average, after the addition of 3 µM CGS7184, the cytoplasmic calcium concentration rapidly increased, up to approximately 160% of the resting state, and remained elevated for the rest of the measurement time (Figure 5B). Next, we performed similar experiments using a calcium-free medium (panel A, “−Ca^2+^” and panel C). After the addition of 3 µM CGS7184, we observed a slow increase in the cytoplasmic calcium concentration; however, this was transient and was followed by a drop to the initial value after 3–4 min (Figure 5C). These experiments suggest that CGS7184 can induce calcium influx not only from extracellular medium, but also calcium release from internal calcium stores (e.g., the endoplasmic reticulum).

### 2.5. Calpain Activation Mediates CGS7184 Toxicity

**Figure 6:**
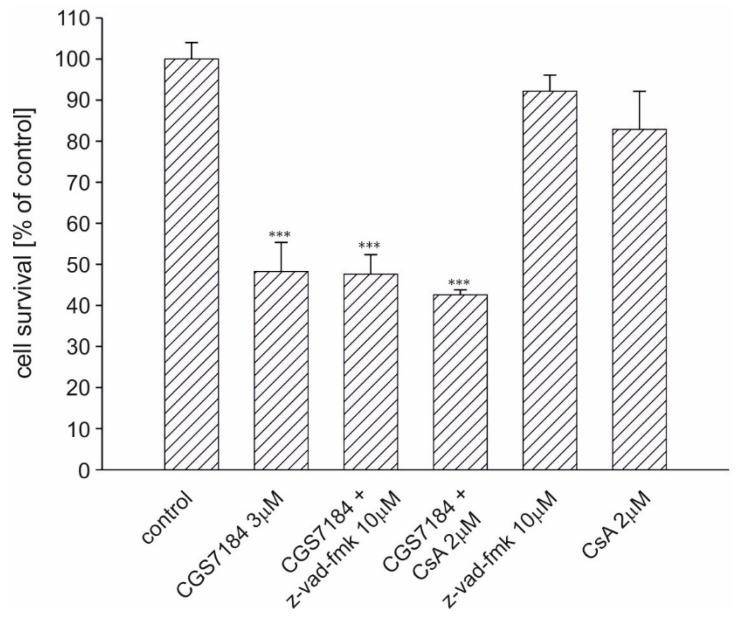
Toxicity induced by CGS7184 is not diminished by the inhibitor of PTP (CsA) or caspases (z-vad-fmk). HT22 cells were treated with the potassium channel opener CGS7184, the inhibitor of PTP (CsA) or caspases (z-vad-fmk) added alone or in combinations. Cell survival was measured using the MTT assay 18 h after insult. All data are expressed as means ± SEM from three independent experiments with three replicates per data point. *** *p* < 0.001 vs. control by one-way ANOVA followed by Tukey’s test.

We sought to determine which neuronal cell death pathways were activated after CGS7184 treatment. It is known that mitochondria take up excess calcium ions present in the cytoplasm. However, the calcium-buffering ability of mitochondria is limited. Exceeding the threshold of the mitochondrial matrix calcium concentration may result in the induction of permeability transition pore (PTP) and subsequent activation of the mitochondrial apoptotic pathway, which involves the increased activity of caspases. We therefore tried to block the CGS7184 toxicity with the PTP blocker, cyclosporine A, and the pan-caspase inhibitor z-vad-fmk (Figure 6). HT22 cells were preincubated for 30 min with blockers, after which 3 µM CGS7184 was added. Cell survival was measured after 18 h of incubation with CGS7184. We found that neither CsA at 2 µM nor z-vad-fmk at 10 µM blocked CGS7184-induced toxicity.

**Figure 7:**
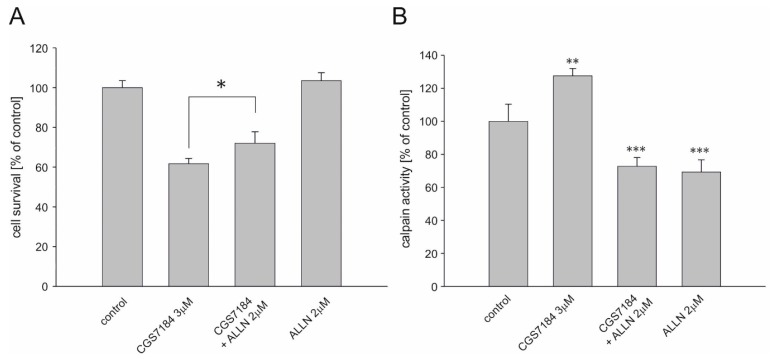
Toxicity induced by CGS7184 involves the activation of calpains. (**A**) HT22 cells were treated with 3 µM CGS7184 alone or in the presence of 2 µM calpain inhibitor I (ALLN). Cell survival was measured using the MTT assay 18 h after insult. (**B**) HT22 cells were treated with 3 µM CGS7184 alone and in the presence of 2 µM calpain inhibitor I (ALLN). Calpain activity was measured using the Calpain-Glo assay 30 min after treatment. All data are expressed as means ± SEM from three (**A**) and four (**B**) independent experiments with three replicates per data point. * *p* < 0.05, ** *p* < 0.01, and *** *p* < 0.001 by one-way ANOVA followed by Tukey’s test.

The increased level of cytoplasmic concentration of Ca^2+^ ions could also activate the calpain protease system, which represents an alternative cell death pathway potentially induced during CGS7184 treatment. To determine whether this was the case, we measured cell survival in the presence of different calpain inhibitors. We found that the toxicity induced by 3 µM CGS7184 was attenuated by 2 µM ALLN (Figure 7A). Similar results were obtained when we used another calpain inhibitor, EST at 30 µM [28]. Finally, we monitored the activity of calpain in HT22 cells after the addition of CGS7184 (Figure 7B). Thirty minutes of incubation of HT22 cells in the presence of 3 µM CGS7184 resulted in a25% increase of calpain activity. When HT22 cells were preincubated with ALLN for 15 min, then incubated for anadditional 30 min after the addition of 3 µM CGS7184, calpain activity decreased by 77%. Similar results were obtained when ALLN was used alone. It is therefore plausible that CGS7184 toxicity is at least in part due to calpain activation.

## 3. Discussion

In the present study, we investigated the effects of potassium channel openers CGS7181 and CGS7184 in three different experimental systems: single channel recordings of mitoBK_Ca_, isolated brain mitochondria/brain homogenate, and in vitro cell culture.

Potassium channel openers have gained attention mainly as new therapeutic agents that could be used to prevent cell death that occurs after insults such as ischemia/reperfusion. Many studies have shown that the application of KCOs protects different tissues both in vitro and in vivo. Although the mechanism of cytoprotection is still unclear, it was suggested that mitochondrial potassium channels play a crucial role in this phenomenon [7,17]. On the other hand, a growing number of data revealed non-specific interactions of potassium channel modulators, indicating that these compounds may influence cellular and mitochondrial function independently of their primary targets [1].

Here, we described both specific and unspecific effects of tested KCOs. Using single channel recordings, we showed for the first time that CGS7184 activates the mitochondrial BK_Ca_ channel in human astrocytoma cells. A channel-related effect was also observed when CGS7184 was applied to isolated rat brain mitochondria and rat brain homogenate. These results support previous observations describing this drug as a potassium channel opener. First, it was directly shown that CGS7184 increases the activity of BK_Ca_ channels from the plasma membrane [3]. Second, our previous study showed, indirectly, that CGS7184 can activate the mitoBK_Ca_ from brain mitochondria. In this case, channel activation was manifested by reduced reactive oxygen species synthesis by rat brain mitochondria and this effect was attenuated by BK_Ca_ channel blockers [16]. Current experiments showed that activation of the mitoBK_Ca_ channel by the application of CGS7184 induces an influx of potassium ions to the negatively charged matrix and promotes mild uncoupling of mitochondria. This stimulates activity of the mitochondrial respiratory chain to restore mitochondrial membrane potential by pumping protons from the matrix to the mitochondrial intermembrane space. Increased activity of respiratory chain is manifested by increased oxygen consumption by cytochrome c oxidase (terminal complex of respiratory chain), even in the absence of additional ADP. Observed effects induced by CGS7184 were potassium and charybdotoxin dependent, which indicates specific involvement of the mitoBK_Ca_ channel.

However, when used in the HT22 cell line, CG7181 and CGS7184 exhibited strong cytotoxic effects. In contrast, other BK_Ca_ channel openers, NS1619 and NS004, did not show any toxicity even at a higher concentration (30 μM), which is in line with the previous studies in which the application of NS1619 at a concentration of up to 150 μM did not induce a cytotoxic effect in neuronal cultures [18]. Cell death induced by CGS7184 seems to be unrelated to the activation of BK_Ca_ channels, since the application of BK_Ca_ channel inhibitors did not block the CGS7184-induced toxic effect. Previous studies showed that cytoplasmic potassium efflux can promote neuronal apoptosis [29] and the inhibition of potassium channels from the neuronal plasma membrane can be cytoprotective. For example, such an effect was observed when potassium-selective ion channel blockers like tetraethlyammonium ions were used [30,31]. Similar cytoprotection was reported when K_ATP_ channel inhibitors were applied [32,33]. However, our previous study revealed that paxilline protects HT22 cells against glutamate toxicity without affecting the BK_Ca_ channel activity [34].

Interestingly, some potassium channel openers may modulate functions of mitochondria by mechanisms not related to the activation of mitochondrial ion channels, e.g., NS1619 inhibits respiratory chain activity in glioma cells [20]. Later studies also revealed that this activator can interact with mitochondria to induce mitochondrial depolarization without affecting mitoBK_Ca_ channels and subsequently induce cytoprotection [18,19]. What is more, NS1619 was shown to inhibit the sarco/endoplasmic reticulum calcium pump (SERCA), mitochondrial complex I, and ATP synthase [35].

In our current study, we discovered that the non-specific effects of CGS7184 are related to the deregulation of calcium homeostasis. Precise control of the Ca^2+^ ion concentration in the cytoplasm is crucial for proper cell function. We observed a rapid increase in cytoplasmic Ca^2+^ levels after the addition of CGS7184. This effect was observed in the presence or absence of Ca^2+^ in the experimental medium. This may suggest that CGS7184 promotes calcium release from intracellular stores such as the endoplasmic reticulum and is in line with previous findings that CGS7184 and NS1619 induce calcium release from intracellular stores in endothelial and muscle cells [21,22,23]. It has been shown that CGS7184 increases the open probability of the ryanodine channel from isolated sarcoplasmic reticulum [22] and in consequence promotes calcium release from the sarcoplasmic reticulum to the cytoplasm.

Usually, mitochondria can buffer excessive cytosolic Ca^2+^ levels by transport into the matrix. However, the uncontrolled calcium influx into mitochondria induces the opening of PTPs and the leakage of cytochrome c into the cytoplasm. This leads to caspase activation and apoptosis [36,37,38,39]. We used cyclosporin A to prevent mitochondrial PTP opening and z-vad-fmk, a cell-permeant pan-caspase inhibitor that irreversibly binds to the catalytic site of caspase proteases to inhibit the induction of apoptosis. None of these drugs blocked CGS7184-induced toxicity. These observations suggested that alternative pathways are critical for inducing CGS7184 toxicity. One of such alternative pathways might be the direct activation of calpain proteases by elevated levels of Ca^2+^. The involvement of calpains in cell death is well documented [40,41,42,43,44]. It has been shown that calpains cleave a variety of proteins to promote apoptosis. In our experiments, calpain inhibitors partially blocked CGS7184-induced cell death. Although the classical apoptosis pathway includes the activation of caspases, we did not observe attenuation of CGS7184-induced toxicity after the application of a caspase inhibitor. Furthermore, we were able to show direct calpain activation after CGS7184 stimulation using a luminescence assay. These results support the hypothesis that the KCO-induced toxicity is mediated by calpain activation. The similar effect of CGS7184 was observed in human glial cells [23]. However, it has to be mentioned that we observed only partial relief of CGS7184 induced cytotoxicity by the calpain inhibitor. This might suggest that alternative cell death pathways might also be activated. Another interesting question is the exact role of calpain proteases in the cytotoxicity we observed in light of the fact of the recent identification of calpains in brain mitochondria [45]. Additionally, it is possible that calpains are directly activatated by CGS7184. All these issues require further detailed investigation.

Finally, the application of higher concentrations of CGS7181 or CGS7184 (30µM) resulted in very fast and massive cell death. This might suggest that both compounds interact with cellular membranes. We observed that the addition of >3 µM CGS7184 usually resulted in unstable recordings [46], indicating a possible interaction of this drug with membranes, which is consistent with the high logP of this molecule (4.52-predicted with ALOGPS 2.1, http://www.vcclab.org/lab/alogps/). This is also consistent with a relative high variability of the CGS7184 effect on mitoBK_Ca_ channel activity seen in patch-clamp experiments, since there is no defined binding site and a variable number of molecules could partition into membranes depending on the time of incubation, membrane area, and local lipid composition, etc. However, this issue still requires a more detailed study.

In summary, we have shown that the potassium channel opener CGS7184 activates mitoBK_Ca_ channels. However, in parallel, this compound induces neuronal cell death, likely by increasing cytoplasmic calcium. The cytotoxic effects of this compound were independent of BK_Ca_-type channel activation, clearly showing pleiotropic sites of action of these chemicals in neuronal cells.

## 4. Materials and Methods

### 4.1. Materials

Chemicals for cell cultures were purchased from Life Technologies (Carlsbad, CA, USA) (Gibco) and Sigma-Aldrich Co., Ltd. (St. Louis, MI, USA) The NS1619 was from Sigma-Aldrich Co., Ltd.; CGS7181 and CGS7184 were a kind gift from Dr. Michele Chiesi at Novartis Pharma. The calpain activity detection kit was from Promega (Madison, WI, USA). The Fura 2-AM was from Molecular Probes (Eugene, OR, USA). BK_Ca_ channel inhibitors (iberiotoxin and charybdotoxin) were from Alomone Labs (Jerusalem, Israel); paxilline was from Biomol (Hamburg, Germany). Calpain and caspase inhibitors were purchased from Calbiochem (a brand of EMD Biosciences Inc., La Jolla, CA, USA). Cyclosporin A was purchased from Alexis Biochemicals (Lausen, Switzerland). All other chemicals were of the highest purity available commercially and, unless otherwise indicated, were obtained from Sigma-Aldrich Co., Ltd.

### 4.2. Electrophysiology

Patch-clamp experiments using mitoplasts were performed as previously described [24]. Patch-clamp pipettes were filled with an isotonic solution containing 150 mM KCl, 10 mM HEPES, and 100 µM CaCl_2_ at pH  7.2. The low-calcium bath solution (1 µM CaCl_2_) containing: 150 mM KCl, 10 mM HEPES, 1 mM EGTA, and 0.752 mM CaCl_2_ at pH   7.2, was used to record channel activity in control conditions and in the presence of CGS7184 and paxilline. Modulators were added directly with mixing to the recording chamber. The experiments were carried out in the inside-out mode with the mitochondrial matrix facing the bath solution. The voltages applied to the patch-clamp pipette interior are reported. Currents were recorded using a patch-clamp amplifier (Axopatch 200B, Molecular Devices Corporation, Sunnyvale, CA, USA). The pipettes were made of borosilicate glass and had a resistance of around 10 MΩ. The currents were low-pass filtered at 1 kHz and sampled at a frequency of 10 kHz. The probability of the channel being open, *NP*_o_, was determined using the Clampfit 10.2 software routine according to the formula:

(1) $$NP_{o}=(\sum_{j=1}^{N}t_{j}j)/T$$

where *P*_o_ is the single-channel open-state probability; *T* is the duration of the measurement; *t_j_* is the time spent with *j* = 1, 2, …, *N* channels open; and *N* is the maximal number of simultaneous channel openings seen in the patch.

### 4.3. Rat Brain Mitochondria Isolation

Animal protocols were in accordance with guidelines for the humane treatment of animals and were reviewed and approved by the Animal Ethics Committee of North Rhine-Westphalia, Germany.

Rat mitochondria were isolated according to the protocol described previously [16]. Briefly, one brain (without cerebella) from a ~50 days old male Wistar rat was minced and homogenized in an ice-cold MSE medium (225 mM mannitol, 75 mM sucrose, 1 mM EGTA, 5 mM HEPES, 1 mg/mL essential fatty acid free BSA, pH = 7.4) containing 0.5 mg/mL of bacterial protease nagarse (Fluka, Neu-Ulm, Germany). The homogenate was centrifuged for 4 min at 2000× *g* at 4 °C. Obtained supernatant was centrifuged for 9 min at 12,000× *g* at 4 °C. Resulting pellet was homogenized in MSE medium containing 0.2 mg/mL of digitonin (Fluka, Germany) and centrifuged for 11 min at 12,000× *g* at 4 °C. The pellet (mitochondrial preparation) was resuspended in MSE medium.

### 4.4. Rat Brain Homogenate Preparation

A brain (without cerebellum) of a sacrificed male Wistar rat (~50 days old) was rapidly removed, washed, and immediately placed into the ice-cold MSE medium. About 200 mg of wet tissue was homogenized on ice twice for 20 s at 8000 rpm using an Ultra-Turrax T 25 homogenizer (IKA, Staufen, Germany) in 0.5 mL MSE medium.

### 4.5. Oxygen Consumption Rate

Oxygen consumption rate measurements were performed in an air-saturated buffer (10 mM KH_2_PO_4_, 60 mM KCl, 60 mM Tris, 110 mM mannitol, 0.5 mM EDTA, pH = 7.4) at 30 °C with the use of an Oxygraph-2k (Oroboros, Austria). In each measurement, around 175 µg of isolated rat brain mitochondria preparation was mixed with 2.0 mL of buffer containing 5 mM malate, 10 mM glutamate, and 5 mM MgCl_2_. The state III respiration was induced by the addition of 2 mM ADP.

### 4.6. Cell Culture

Astrocytoma U-87 MG cells and HT22 cells were grown in Dulbecco’s modified Eagle’s medium (DMEM) supplemented with 10% fetal bovine serum (FBS), 100 units/mL penicillin, 100 μg/mL streptomycin, and 4 mmol/L l-glutamine. Cells were incubated in a humidified 5% CO_2_ atmosphere at 37 °C.

### 4.7. MTT Cell Viability Assay and Lactate Dehydrogenase Assay

Cell viability was assessed by measuring the ability to metabolize 1-(4,5-demethyldiazol-2-yl)-2,5-diphenyltetrazolium bromide (MTT). HT22 cells were seeded onto 96-well plates at a density of 5 × 103 cells per well, with each well containing 100 μL medium. After incubation, cells were treated with chemicals as indicated in the figure legends. Following the indicated treatments, experimental medium was replaced with 50 μL of MTT (0.5 mg/mL) solution in DMEM and incubated for 2–3 h. Then, 50 μL of lysis buffer containing 20% (*w*/*v*) SDS, 50% (*v*/*v*) DMF, 2% (*v*/*v*) acetic acid, and 25 mM HCl was added. The changes in absorbance of formazan dye were measured at 570 nm using a microplate reader (Tecan, Männedorf, Switzerland), with a reference at 655 nm. Lactate dehydrogenase (LDH) enzyme activity was assayed using a commercial kit (Roche Molecular Biochemicals, Basel, Switzerland). The results are expressed relative to the controls specified in each experiment. Values are expressed as the mean ± SEM of at least three independent experiments.

### 4.8. Fluorescence Measurements of Ca^2+^ Concentration in Single Cells

HT22 cells were seeded on coverslips treated with poly-l-lysine (10 μg/mL). After 24 h, the cells were loaded with the fluorescent dye Fura 2-acetoxymethyl ester (Fura 2-AM; Molecular Probes) at 2 μM for 30 min at 37 °C in DMEM. After loading, the HT22 cells were washed with Krebs-HEPES buffer containing (in mM): 120 NaCl, 4.75 KCl, 1.2 MgSO_4_, 25 NaHCO_3_, 1.2 KH_2_PO_4_, 1.2 CaCl_2_, 5 glucose, and 25 HEPES. Cells were then mounted in a chamber attached to the stage of an inverted microscope (IX 70, Olympus, Tokyo, Japan), which was equipped with a 20× objective. Fluorescence was visualized at excitation wavelengths of 340 and 380 nm and an emission wavelength of 550 nm with a Till Photonics system equipped with a monochromator (Polychrome IV, Till Photonics, Gräfelfing, Germany). All measurements were carried out at room temperature. The results obtained were analyzed with a TillVision system (TillPhotonics, Graefelfing, Germany).

### 4.9. Calpain Activity Measurements

Calpain activity in HT22 cells was measured using the calpain–Glo assay (Promega). HT22 cells were seeded onto 96-well plates at a density of 5 × 10^3^ cells per well, with each well containing 100 μL medium. Twenty-four hours after plating, proluminescent calpain substrate, Suc-LLVY-aminoluciferin, was added to a final concentration of 20 μM. After 30 min of incubation, 3 μM CGS7184 was added. Thirty minutes later, cells were lysed with buffer containing 0.9% Triton-X 100 in PBS with 50 μM ALLN to block calpain activity. Next, 25 μL of a preparation of Calpain-Glo luciferase detection reagent in Calpain Glo buffer was added to detect free aminoluciferin. Ten minutes after adding the detection reagent, luminescence was measured using a TD-20/20 luminometer (Turner Design).

## 5. Conclusions

Our data show that CGS7184 activates the mitoBK_Ca_ channels from brain tissue. However, both CGS7181 and CGS7184 also induce cell death when used in cellular model. Observed cytotoxic effects are most likely related to the deregulation of calcium homeostasis by studied potassium channel openers followed by calpain proteases activation.

## Abbreviations

IbTx	Iberiotoxin
Pax	Paxilline
KCOs	Potassium channel openers
PTP	Permeability transition pore
